# Evaluation of MMV Pandemic Response Box compounds to identify potent compounds against clinically relevant bacterial and fungal clinical isolates *in vitro*

**DOI:** 10.1016/j.nmni.2024.101444

**Published:** 2024-06-20

**Authors:** Seshan Sivasankar, Appalaraju Boppe, Martin Peter Grobusch, Sankarganesh Jeyaraj

**Affiliations:** a PSG Center for Molecular Medicine and Therapeutics, PSG Institute of Medical Sciences and Research, Coimbatore, India; b PSG Center for Genetics and Molecular Biology, Off Avinashi Road, Coimbatore, India; c Department of Microbiology, PSG Institute of Medical Sciences and Research, Coimbatore, India; d Center of Tropical Medicine and Travel Medicine, Department of Infectious Diseases, Amsterdam University Medical Centers, Location Amsterdam, Amsterdam, the Netherlands; e Infection and Immunity, Amsterdam Public Health, University of Amsterdam, Meibergdreef 9, 1105 AZ, Amsterdam, the Netherlands; f Centre de Recherches Médicales de Lambaréné CERMEL, Hospital Albert Schweitzer, BP 242, Lambaréné, Gabon; g Institut für Tropenmedizin, Eberhard Karls Universität Tübingen and German Center for Infection Research (DZIF), Tübingen, Germany; h Masanga Medical Research Unit, Masanga, Sierra Leone; i Institute of Infectious Diseases and Molecular Medicine, University of Cape Town, Cape Town, South Africa

**Keywords:** MMV compounds, Bacterial and fungal MDR clinical isolates, Epetraborole, Eravacyclin*e*, Oteseconazole, Alexidine, Persister assay

## Abstract

**Background:**

Multidrug resistant bacterial and fungal pathogens are resistant to a number of significant front-line drugs, hence, identification of new inhibitory agents to combat them is crucial. In this study, we aim to evaluate the activity of Pandemic Box compounds from Malaria Medicines Venture (MMV) against *A. baumannii* and *P. aeruginosa* bacterial, *C. auris, C. albicans and A. niger* fungal clinical isolates.

**Methods:**

Isolates were initially screened with 201 antibacterial and 46 antifungal compounds (10 μM) using a microbroth dilution in triplicates to determine MIC. A persister assay was performed for bacterial pathogens.

**Results:**

Out of 201 antibacterial compounds, twenty-nine and seven compounds inhibited the growth of *A. baumannii* and *P. aeruginosa* at 10 μM, respectively. MMV1580854, MMV1579788, eravacycline and epetraborole inhibited both the bacterial test isolates. In a persister assay, MMV1634390 showed complete bactericidal effect against *A. baumannii*. With antifungal activity compounds, *C. auris* responded to15 compounds, Six compounds inhibited *C. albicans* and one was effective against *A. niger* at 10 μM. The ratio of Minimum Fungicidal Concentration (MFC): Minimum Inhibitory Concentration (MIC) of MMV1782110 was 2 against *C*. *auris*. Eberconazole, amorolfine and luliconazole are fungicidal targeting *C. albicans* at a MFC: MIC ratio of 2.

**Conclusion:**

Five compounds from MMV Pandemic Box were found to be inhibiting colistin and ceftazidime resistant *A. baumannii* clinical isolate, also against colistin and β-lactam resistant *P. aeruginosa* clinical isolate*.* MMV1634390 showed complete bactericidal effect against *A. baumannii* in a persister assay. MMV1782110, Eberconazole, amorolfine and luliconazole exhibited potent anti-fungal activity. Further investigations are warranted to identify the targets and mechanism.

## Highlights

• MMV Pandemic Response Box compounds were evaluated against MDR bacterial and fungal pathogens isolated from patients.
• MMV1634390 compound was highly bactericidal against *A. baumannii*, whereas eravacycline and epetraborole were against both *A. baumannii* and *P. aeruginosa.*
• Few compounds were fungicidal against MDR *Candida* and *Aspergillus* fungal species.
• A novel compound MMV1782110 showed excellent inhibitory activity against *Candida auris*.

## 1 Introduction

The slow pace of new antibiotic discovery and the rapid emergence of drug-resistant pathogens imposts a significant burden onto human health. Apart from community-acquired infections, individuals with a compromised immune system, particularly with HIV/AIDS, those with severe burns, patients receiving chemotherapy, and neonates are at higher risk of developing co-morbidities due to bacterial and fungal infections [1,2].

Multidrug-resistant (MDR) *A. baumannii* and *Pseudomonas aeruginosa* are the most-common causative agents of infections including bacteraemia, pneumonia, meningitis, and urinary tract infections [3,4]. MDR pathogens are associated with prolonged hospital stays and higher cost alongside with high morbidity and mortality [3,4].*A. baumannii and P. aeruginosa* are gram-negative non-fermentative bacteria belonging to the Moraellaceae and Pseudomonadaceae families, respectively [3,4]. Both organisms are members of the ESKAPE pathogen family, which poses a serious threat to human health, acquiring antimicrobial resistance at a constant rate [4]. *A. baumannii* has the ability to survive on dry surfaces, and is partially resilient to disinfectants, constituting a significant cause for nosocomial transmissions [5].*A. baumannii* and *P. aeruginosa* are intrinsically resistant to most antibiotics such as fluoroquinolones, tetracyclines, β-lactams, aminoglycosides and carbapenems [4,6]. Concurrently, they emerge as multi- or pan-drug resistant strains; also against colistin, which is being considered the last resort for successful treatment [7]. A number of resistance mechanisms such as target modification, limited permeability of the cell wall, increased activity of efflux pumps and antibiotic hydrolysing enzymes that render antibiotics ineffective have been documented [3,4]. Biofilm formation leads to cross resistance due to low penetration and plays a vital role in the development of preliminary antibiotic resistance mechanisms in *A. baumannii* and *P. aeruginosa* [5,8]. The World Health Organization classified carbapenem resistant *A. baumannii* and carbapenem resistant *P. aeruginosa* as critical pathogens that require urgent research and development for new drug molecules [9].

Recently, WHO has released a fungal priority pathogen list which include *Candida* and *Aspergillus* spp. with a high mortality rate of 40 % [10] and 40–90 % [11],respectively. *Candida* and *Aspergillus* species are among the etiological agents causing invasive fungal infections (IFIs) [12]. Although *Candida albicans* is the major causative agent of invasive candidiasis, other *Candida* species are also acting as the causative agent of candidiasis, especially *C. auris* [13].*C. auris* causes major outbreaks in hospitals and clinical facilities after its first report of an ear infection in Japan 2006. Contrary to *C. albicans*, *C. auris* can reside on human skin for weeks, even after treatment and has the ability to survive on external surfaces [14]. According to CDC's 2019 report, *C. auris* is one among five urgent threats [15]. *A. niger* is not frequently reported in invasive disease, but it is seen causing otomycosis, cutaneous infections, and pulmonary infections with high fatality [16]. In everyday clinical settings, antifungal susceptibility testing has become a routine diagnostic method mainly due to increasing *in vitro* resistance of these pathogens [12]. There have been several reports in the recent years describing azole and echinocandin and azole resistant clinical isolates [12]. Pan drug-resistant (PDR) *C. auris*, resistant to all three classes of antifungals, has also been documented in a number of different countries [17]. To mitigate the emergence and treatment of these fungal pathogens, it is necessary to develop new drugs.

We set out to test the Pandemic Box compounds from Medicines for Malaria Venture (MMV) against multi drug-resistant (MDR) *A. baumannii* and *P. aeruginosa* bacterial clinical isolates and *C. albicans, C. auris* and *A. niger* MDR fungal clinical isolates. MMV and Drugs for Neglected Diseases initiative (DNDi), in collaboration with scientists in academia, provide open-source drug discovery compounds. Researchers from all over the world have studied the 400 structurally different compounds in the Pandemic Response Box in an effort to combat bacterial, fungal, parasitic and viral pathogens that cause a variety of infectious and neglected tropical diseases [18]. The purpose of this study is to evaluate the inhibitory potential of 201 antibacterial and 46 antifungal compounds from the MMV pandemic box library against the mentioned bacterial and fungal clinical isolates. Persister bacterial cells, that remain dormant during initial antibiotic exposure, were determined after the drug treatment for bacterial samples. Fungicidal concentrations of the drug molecules were assessed.

## 2 Materials and methods

### 2.1 Bacterial strains and growth conditions

Colistin-resistant clinical isolates of *A. baumannii* and *P. aeruginosa* were collected from the NABL and NABH accredited Microbiology Diagnostic Laboratory of the PSG Institute of Medical Sciences and Research, Coimbatore, India. *A. baumannii* was isolated from a hemodialysis catheter of the right external jugular vein of a patient with a history of chronic kidney disease. *P. aeruginosa* was isolated from the abdominal pus of a patient presenting with abdominal abscesses. Catheter and pus swabs were rubbed onto the blood agar and incubated at 37 °C overnight. Following incubation, colonies that emerged were streaked onto MacConkey agar (Himedia, Mumbai, India) plates supplemented with colistin (SRL, Mumbai, India) (4 μg/ml, in accordance with CLSI guidelines) [19]. The colonies were then incubated at 37 °C for 24 h and subsequently stored at 2 °C until needed. Bacteria were initially identified using Biochemical tests andVitek2®GN ID card. Antibiogram testing was performed using Kirby-Bauer method and Vitek2® AST card N280 following CLSI guidelines 2021(BioMérieux, Marcy-l'Étoile, France). The colistin disc broth elution (CDBE) assay was performed to identify resistance amongst test clinical isolates against colistin according to CLSI guidelines. This CBDE assay was performed as previously described by Simner et al., 2023 [20]. Briefly, this assay involves four tubes with colistin discs of 0,1, 2 and 4 μg/ml concentration of colistin, respectively. Fifty μl of the inoculum were added to each tube to obtain a final concentration of 5 × 10^5^ CFU/ml in accordance with CLSI guidelines. The growth of the organisms was assessed visually after incubation. Growth controls consisted of tubes inoculated with test pathogens, while the *E. coli* ATCC 25922 strain with colistin disc acted as negative control. The MIC of the test strains was determined using commercial MIKROLATEST MIC®-MIC colistin kits (ErbaLachemas.r.o, Brno, and Jihomoravskykraj, Czech Republic). CAMHB was used for the bacterial screening and assays.

### 2.2 Fungal strains and growth conditions

*C. albicans*, *C. auris* and *A. niger* fungal strains were isolated from patient samples. Germ tube test, lactophenol cotton blue staining methods were used for preliminary identification of the fungal species and were confirmed withVitek2® using YST ID card. Antifungal sensitivity testing was performed using Vitek2®. *Candida* strains were inoculated into 5 ml YPD media (1 % yeast extract; 2 % peptone; 2 % dextrose; 0.5 % ammonium sulphate) and incubated overnight at 35 °C (150 rpm) prior to use. Fresh *A. niger* culture on Sabouraud dextrose agar (SDA) slant (Himedia, Mumbai, India) was used for the assay. YPD was used for all fungal screening assays such as preliminary screening, MIC, and MFC determination assays.

### 2.3 DNA extraction, species confirmation and colistin resistant gene

Bacterial DNA was extracted using QIAamp DNA Mini kit (Qiagen, Hilden, Germany) following the manufacturer's instructions. The extracted DNA samples were subjected to 16s rRNA gene PCR amplification, and amplicons were visualized with the agarose gel electrophoresis method. Using the Genetic Analyzer 3500, amplified PCR products were sequenced using the Sanger method for species confirmation (Applied Biosystems Hitachi-High-tech, Tokyo, Japan). Chromatogram and raw reads were visualized using Finch TV and subjected to NCBI BLASTn to match the identity. Presence of *mcr* genes (*mcr1 to mcr5*) and *mgrB gene* alteration were detected using PCR and agarose gel electrophoresis methods. Primer sequences were used from the previously reported studies [21].PCR reaction mix consisted of 7.5 μl of Taq DNA polymerase 2x master mix RED (Ampliqon, Denmark), 200 nM of primers, 1 μl of template and nuclease free water to make up 15 μl PCR volume. PCR conditions was set as follows: 3 min of 94 °C initial denaturation, except for *mcr2* with 15 min of initial denaturation, 30 cycles of 94 °C for 30 s, 90 s of annealing temperature (*mcr1* – 47.9 °C; *mcr2* - 56.5 °C; *mcr3* - 48.4 °C;*mcr4* – 46.8 °C;*mcr5* - 49.5 °C; *mgrB* - 54 °C), and 60 s of 74 °C final elongation followed by termination at 72 °C for 10 min. The amplicons were visualized using agarose gel electrophoresis method using 1.5 % agarose.

### 2.4 MMV pandemic box

The compounds in the MMV Pandemic Response Box were provided in five 96-well microtiter plates, each plate containing 80 compounds. Initially, each well of the plate contained10μl of a 10 mM compound solution in DMSO. As per MMV instructions, 90 μl of DMSO was added to the 10 mM stock solution to dilute it to 1 mM. The first and last columns in each plate were left empty, reserved for negative and positive controls, respectively [18]. All plates were stored at −20 °C, until use. The structure, IUPAC name and cytotoxicity data of the compounds were provided by MMV (Table .4).

### 2.5 Inhibitory activity screening

Bacterial cultures and fungal cultures were inoculated into 5 ml of Cation Adjusted Muller Hinton Broth (CAMHB) and YPD media, respectively, followed by incubation at 37 °C overnight with shaking at 150 rpm. The suspension turbidity of overnight culture was adjusted to an optical density of 0.1 ± 0.02 absorbance at 600 nm or 0.5 McFarland scale, which is equivalent to 1.5 × 10^8^ CFU/ml. In case of *A. niger,* a loopful of conidia was suspended in saline with 1 % Tween-20 and adjusted to 0.5 McFarland. The turbidity-adjusted culture was serially diluted to obtain a 10 × 10^5^ CFU/ml inoculum in corresponding media (M_i_). The inoculum (50 μl) was dispensed into the wells of the 96-well test plate. Three microliters of 1 mM stock compounds (to possess antibacterial activity) were diluted with 147 μl of growth media (M_c_) to a concentration of 20 μM, with 150 μl of compound-broth mixture. Fifty μl of the Pandemic Response Box compounds at a concentration of 20 μM (M_c_) were then dispensed into the wells of 96-well microtiter plates containing 50 μl of 10 × 10^5^ CFU/ml culture (M_i_) (50 μl M_c_+50 μl M_i_), resulting in a final solution with 10 μM concentration of the test compound, 5 × 10^5^ CFU/ml, and 1%DMSO (in triplicates). All the compounds were screened in triplicates. The first and last columns of each plate were representing growth controls (culture without drugs) and blank (only media). The microtiter plates were sealed with optically clear micro sealers and incubated at 37 °C overnight. *A. niger* assay plates were incubated at 27 °C for 72–96 h, whereas C*. albicans* and *C. auris* plates were incubated at 35 °C for 24 h. Photometric readings were obtained by using a Varioskan Flash Multimode reader (Thermo Scientific, Waltham, Massachusetts, USA). Optical densities (OD)were recorded at 600 nm wavelength for bacteria and *Candida* spp. and at 530 nm for *Aspergillus* spp. The solutions in wells with little or no turbidity were only considered for the persister assay and fungicidal concentration determination. Fluconazole was used as an internal drug control for the preliminary screening against fungal pathogens. The percentage of growth inhibition was calculated using the formula: % of growth inhibition = 100 × [1- (OD_T_ -OD_B_)/(OD_G_-OD_B_)], where OD_T_ was the optical density of wells treated with drug; OD_G_ represented growth control where wells contain no drugs; and OD_B_ denoted the negative control. The compounds that showed inhibition in this screening method were used for further testing including persister assay and MIC determination.

### 2.6 Assessment of bacterial persister frequency

In order to evaluate the surviving population, liquid culture from wells with little or absence of growth were used for measuring the persister frequency (P*f*). Suspensions from the wells were serially diluted up to 10^−3^, and 100 μl of suspension from the final dilution step was spread over the LB agar media and incubated overnight at 37 °C. Manual colony counting was performed to estimate the number of viable bacteria. The persister population was enumerated by calculating CFU with the colony count of each plate in relation to dilution (colony forming unit (CFU) per ml = no. of. colonies × dilution factor × inoculum volume ^−1^).

### 2.7 Minimum inhibitory concentration

Compounds that inhibited growth of the pathogens in the preliminary screening method were subjected to minimum inhibitory concentration (MIC) determination in sterile polystyrene flat-bottom 96-well plate. The final concentration of compounds in MIC determination ranged from 10 to 0.062 μM using microbroth dilution as mentioned before. Each well had a final cell density of 10^5^ cells/ml, and the plates were incubated at 37 °C for 18–24 h. The least concentration at which fungal growth was inhibited completely (100 %) was defined as MIC.

### 2.8 Fungicidal concentration

MMV indicated compounds having antifungal activity only were used to determine fungicidal activity. The fungicidal concentration was determined by transferring an aliquot of 5 μl from the wells that showed no visible fungal growth, in triplicates, from the previous MIC determination Microdilution assay plate to antibiotic free SDA plate supplemented with 2 % dextrose. The minimum fungicidal concentration (MFC) was determined as the least concentration with no fungal colonies on SDA plate after incubation. MFC:MIC ratio was determined to evaluate the compounds as fungicidal (1–2) and fungistatic (>2) [22].

## 3 Results

### 3.1 Antimicrobial susceptibility and molecular analysis

**Fig. 1:**
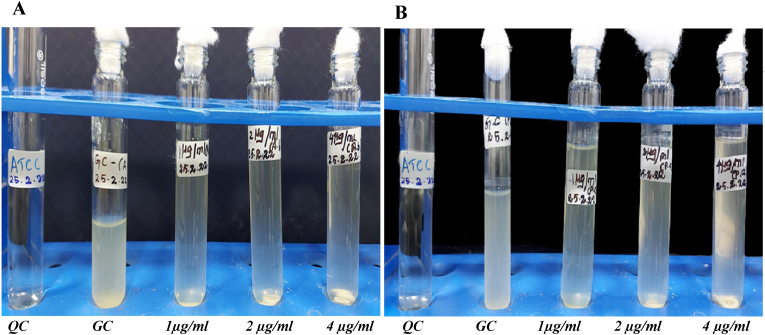
Colistin broth Disk Elution assay with clinical isolates Four tubes for each bacterial strain containing 10 ml of cation adjusted Muller Hinton Broth that generated a final concentration of 0, 1, 2 and 4 μg/ml colistin, respectively. Fig. 1A represents the test for *A. baumannii* colistin resistant strains whereas Fig. 1B displays *P. aeruginosa* growth until 4 μg/ml concentration with ATCC *E. coli* as quality control QC. GC represents growth control tubes with test isolates without colistin drugs.

Turbidity was observed visually in all the tubes containing 0, 1, 2 and 4 μg/ml of final colistin concentration, indicating growth in all tubes (Fig. 1A and B), consistent with CLSI standards. Turbidity was observed for *A. baumannii* and *P. aeruginosa* up to 16 and 8 μg/ml, respectively, in the MIC determination assay using the Microlatest® MIC colistin kit (ErbaLachema, Brno, Czech Republic).*A. baumannii* was found to be resistant to colistin and ceftazidimein the antibiogram tests (Supplementary Table 1). *P. aeruginosa* isolate was resistant to colistin along with most of the β-lactam antibiotics, but sensitive to aztreonam (Supplementary Table 1). *C. auris* strain was resistant to amphotericin B and fluconazole, whereas *C. albicans* was resistant to ketoconazole, fluconazole, clotrimazole (Supplementary Table 2). Sanger sequencing of the 16S region and BLASTn analysis confirmed the clinical isolates as *A. baumannii* and *P. aeruginosa*, with 98.7 % and 99.8 % of identity, respectively. *mcr1-5*genes were found to be absent in both the isolates via PCR method. Test isolates were found to be carrying wild type *mgrB* gene.

### 3.2 Inhibitory activity against bacterial pathogens

In case of *A. baumannii*, 29 drugs inhibited 90–100 % of growth; 15 compounds inhibited 70–89 % of growth; 21 compounds showed 50–69 % of inhibition; and 136 drugs had less than 49 % inhibition effect on growth. *P. aeruginosa* was inhibited actively by a smaller number of drugs, where only seven drugs inhibited the growth completely (90–100 %); two drugs with 70–89 % inhibitory activity; five drugs with 50–69 % of inhibition; and 187 drugs inhibited the growth of target organism less than 49 % (Table 1). More than 75 % of compounds showed less than, or equal to, 50 % growth inhibition at the given concentration on*P.aeruginosa.*MMV1580854, MMV1579788, MMV1578574 and MMV1578566 compounds showed strong inhibitory activity (∼80 %) against both the strains. The compounds that showed more than 90 % inhibitory action against the pathogens were subjected to persister screening.

**Table 1:** Initial screening of bacterial pathogens and persister cells produced after treatment.

Compound	*A. baumannii*		*P. aeruginosa*	
	% of growth inhibition	Persistercount	*% of growth inhibition*	Persistercount
MMV1582497	100.00	TNTC		
MMV1581548	99.16	TNTC		
MMV1578568	100.00	TNTC	96.66	1 × 10^−1^ CFU/ml
MMV1580173	93.04	TNTC		
MMV1634390	100.00	No colonies		
MMV1581552	54.87	TNTC		
MMV1580853	96.10	TNTC	60.69	TNTC
MMV1580841	50.70	TNTC		
MMV508427	100.00	TNTC		
MMV1634383	76.04	TNTC		
MMV1580848	56.55	TNTC		
MMV1580840	70.47	TNTC		
MMV1579846			97.18	TNTC
MMV1578842	97.21	TNTC		
MMV1579354	79.94	TNTC		
MMV1578571	96.10	5.5 × 10^−2^ CFU/ml		
MMV1633966	78.55	TNTC		
MMV002459	95.54	3.8 × 10^−2^ CFU/ml	100.00	TNTC
MMV637945	59.05	TNTC		
MMV1580854	96.66	TNTC	55.27	TNTC
MMV1580851	91.09	TNTC		
MMV1579849	97.77	TNTC	60.17	
MMV020752	100.00	TNTC		
MMV1578575	97.21	TNTC		
MMV1663457	83.84	TNTC		
MMV093250	100.00	TNTC		
MMV1593541	99.16	2.4 × 10^−2^ CFU/ml		
MMV1593537	97.21	TNTC		
MMV1582492	90.25	TNTC		
MMV1582491	96.10	TNTC	60.79	TNTC
MMV1580844			90.8	TNTC
MMV1579788	84.40	TNTC	93.95	TNTC
MMV1579785	94.43	TNTC		
MMV1578559	58.22	TNTC		
MMV1578574	92.20	TNTC	98.75	6.3 × 10^−1^ CFU/ml
MMV1578566	92.48	TNTC	97.08	1.7 × 10^−2^ CFU/ml
MMV1782140	72.14	TNTC		
MMV1634391	83.57	TNTC		
MMV1633674	57.38	TNTC		
MMV1593532	87.19	TNTC		
MMV637659	79.11	TNTC		
MMV1579850			73.10	TNTC
MMV374187			52.24	TNTC
MMV688756	52.37	TNTC		
MMV1578897	67.13	TNTC		
MMV001014	51.25	TNTC		
MMV1578554	61.00	TNTC		
MMV1634402	87.47	TNTC	79.67	TNTC
MMV000051	76.60	TNTC		
MMV1582496	67.69	TNTC		
MMV1582490	98.05	1.0 × 10^−3^ CFU/ml		
MMV1582488	92.48	TNTC		
MMV002665	50.42	TNTC		
MMV1581559	51.81	TNTC		
MMV1581545	77.16	TNTC		
MMV1580855	61.56	TNTC		
MMV1580849	100.00	3.5 × 10^−2^ CFU/ml		
MMV1580839	65.18	TNTC		
MMV1579778	53.48	TNTC		
MMV099714	48.47	TNTC		
MMV1578891	53.20	TNTC		
MMV124656	50.42	TNTC		
MMV975972	55.71	TNTC		
MMV1578884	97.49	1.9 × 10^−2^ CFU/ml		
MMV108465	71.59	TNTC		
MMV1578570	96.66	9.3 × 10^−2^ CFU/ml		
MMV1578564	96.38	TNTC		
MMV1782403	93.31	6.1 × 10^−2^ CFU/ml		
MMV1613563	54.87	TNTC		

MMV pandemic response box compounds that showed inhibition were mentioned and expressed as percentage (%) of growth inhibition. Percentage of inhibition was calculated using the OD at 600 nm.Persisterfrequencies with respect to each hit compounds against bacterial pathogens arerepresented as CFU/ml (Persister count). * TNTC – too numerous to count.

### 3.3 Bacterial persister frequency

Plates spread with suspension of the wells with 50 % growth inhibition showed colonies that were too numerous to count (TNTC). Liquid culture of complete inhibition wells of the test organisms when spread on LB agar plates produced countable or lesser numbers of colonies (Table 1). Interestingly, the MMV1634390 compound which showed complete inhibition of *A. baumannii* in microbroth dilution was found to be bactericidal with zero persisters. The remainder of the hit compounds against *A. baumannii* were enumerated for persister frequency; as follows, MMV1578571 (5.5 × 10^2^ CFU/ml), MMV002459 (3.8 × 10^2^ CFU/ml), MMV1593541 (2.4 × 10^2^ CFU/ml), MMV1582490 (1.0 × 10^3^ CFU/ml), MMV1580849 (3.58 × 10^2^ CFU/ml), MMV1578884 (1.9 × 10^2^ CFU/ml), MMV1578570 (9.3 × 10^2^ CFU/ml) and MMV1782403 (6.1 × 10^2^ CFU/ml) (Table 1). In case of *P. aeruginosa,* MMV1578568 which was inhibiting the survival of the pathogen at 100 % produced a persister frequency of 1 × 10^1^ CFU/ml. MMV1578574 and MMV1578566 resulted in 6.3 × 10^1^ CFU/ml and 1.73 × 10^2^ CFU/ml of persister frequency, respectively (Table 1).

### 3.4 Identification of antifungal compounds

Internal control fluconazole inhibited 99 % growth of *Candida* and *Aspergillus* fungal strains. Amongst 46 compounds tested, antifungal activity (defined as more than 90 % growth inhibition) was reached by 15 compounds against *Candida auris*. These compounds were eberconazole, isavuconazonium, MMV1634386, alexidine, AN-2718, terbinafine, MMV1782110, abafungin, carbendazim, amorolfine, MMV1782212, TSC-INH, MMV1782221, MMV1782218 and olorofim. Six compounds possessed antifungal activity against *C. albicans*, namely eberconazole (%IG = 98 %), MMV1634386 (%IG = 93 %), ketoconazole (%IG = 94 %), terbinafine (%IG = 95 %), amorolfine (%IG = 92 %) and luliconazole (%IG = 93 %). *A. niger* was inhibited with 99 % of growth by alexidine in preliminary screening (Table 2).

**Table 2:** Percentage of growth inhibition of fungal pathogens by various compounds with expected antifungal activity of Pandemic Response box.

MMV ID	Trivial Name	*Inhibition percentage*		
		C.auris	C. albicans	*A. niger*
MMV1634492	Eberconazole	93	98	
MMV1634494	Isavuconazonium	98		
MMV1634386		98	93	
MMV637533	Ketoconazole		94	
MMV002565	Oxfendazole			
MMV396785	Alexidine	96		99
MMV1634363	AN-2718	90		
MMV1634359	Terbinafine	98	95	
MMV1782110		100		
MMV1634493	Abafungin	94		
MMV344625	Carbendazim	95		
MMV1634358	Amorolfine	92	92	
MMV640014	Deferasirox			
MMV1782212		90		
MMV1782227	TSC-INH	99		
MMV1782221		96		
MMV1782224	Luliconazole		93	
MMV1782223				
MMV1782218		97		
MMV1782354	Olorofim	97		

Antifungal compounds that inhibited fungal pathogens were expressed with Inhibition percentage individually for fungal pathogens used in the study. Compounds are represented with MMV ID and trivial name.

### 3.5 Minimum inhibitory concentrations

Drugs like eberconazole, alexidine, AN-2718, carbendazim, amorolfine, MMV1782212, TSC-INH, MMV1782221, MMV1782218 and olorofim exhibited minimum inhibitory concentration of ≥10μMon*C.auris.* MICs of the compounds such as isavuconazonium (≥0.5 μM), MMV1634386 (≥0.5 μM), terbinafine (≥2 μM), MMV1782110 (≥1 μM) and abafungin (≥3 μM) that inhibited *C. auris* in initial screening were reasonable. *C. albicans* was inhibited by eberconazole, terbinafine and amorolfine at a minimum concentration of ≥ 2 μM. Lowest MIC was exhibited against *C. albicans* by MMV1634386 (≥0.2 μM) and ketoconazole (≥0.5 μM) where, luliconazole (≥3 μM) exhibited highest MIC value. Alexidine showed 3 μM of minimum inhibitory concentration on *A. niger* (Table 3).

**Table 3:** Minimum inhibitory concentration and Minimum fungicidal concentration of antifungal compounds calculated for MIC:MFC ratio.

MMV ID	Trivial name	*C.auris*			*C. albicans*			*A. niger*		
		MIC	MFC	MFC:MIC	MIC	MFC	MFC:MIC	MIC	MFC	MFC:MIC
MMV1634492	Eberconazole	≥10	>10	≥1	≥2	≥4	2			
MMV1634494	Isavuconazonium	≥0.5	≥4	8						
MMV1634386		≥0.5	≥2	4	≥0.2	≥4	16			
MMV637533	Ketoconazole				≥0.5	≥5	20			
MMV002565	Oxfendazole									
MMV396785	Alexidine	≥10	>10	≥1				≥3	>10	≥3.3
MMV1634363	AN-2718	≥10	>10	≥1						
MMV1634359	Terbinafine	≥2	≥5	2.5	≥2	≥5	2.5			
MMV1782110		≥1	≥2	2						
MMV1634493	Abafungin	≥3	≥8	2.6						
MMV344625	Carbendazim	≥10	>10	≥1						
MMV1634358	Amorolfine	≥10	>10	≥1	≥2	≥4	2			
MMV640014	Deferasirox									
MMV1782212		≥10	>10	≥1						
MMV1782227	TSC-INH	≥10	>10	≥1						
MMV1782221		≥10	>10	≥1						
MMV1782224	Luliconazole				≥4	≥8	2			
MMV1782223										
MMV1782218		≥10	>10	≥1						
MMV1782354	Olorofim	≥10	>10	≥1						

Minimum inhibitory concentration (MIC) and Minimum fungicidal Concentration (MFC) were denoted as concentration in μM. MIC and MFC ratio (MIC:MFC) values were interpreted as compounds exhibited MIC:MFC ratio of 1–2 were considered as fungicidal and more than 2 as fungistatic. Compounds that exhibited MIC or MFC of more than 10 μM were not considered as potent drug although some demonstrated MIC:MFC ratio of 1.

**Table 4:** Characteristics of pandemic response box compounds showing inhibition against both bacterial and fungal pathogens.

Compound ID	Trivial name	IUPAC name	Structure	Cytotoxicity Cell survival at100μM	MW
MMV1578574	Eravacycline	(4S,4aS,5aR,12aR)-4-(dimethylamino)-7-fluoro-1,10,11,12a-tetrahydroxy-3,12-dioxo-9-[(2-pyrrolidin-1-ylacetyl)amino]-4a,5,5a,6-tetrahydro-4H-tetracene-2-carboxamide		73 %	631.52
MMV1578566	Epetraborole	3-[[(3S)-3-(aminomethyl)-1-hydroxy-3H-2,1-benzoxaborol-7-yl]oxy]propan-1-ol		90 %	273.56
MMV1634390		methyl N-[[3-(ethylcarbamoylamino)-5-(2-methylpyridin-4-yl)isoquinolin-8-yl]methyl]carbamate		68 %	393.4
MMV1578568	Gepotidacin	3-[[4-(3,4-dihydro-2H-pyrano[2,3-*c*]pyridin-6-ylmethylamino)piperidin-1-yl]methyl]-1,4,7-triazatricyclo[6.3.1.04,12]dodeca-6,8(12),9-triene-5,11-dione		96 %	448.5
MMV1634386		(2R)-2-(2,4-difluorophenyl)-1,1-difluoro-3-(tetrazol-1-yl)-1-[5-[4-(2,2,2-trifluoroethoxy)phenyl]pyridin-2-yl]propan-2-ol		3 %	527.4
MMV1634359	Terbinafine	(E)-N,6,6-trimethyl-N-(naphthalen-1-ylmethyl)hept-2-en-4-yn-1-amine		37 %	327.86
MMV396785	Alexidine	1-(2-ethylhexyl)-3-[N-[6-[[N-[N-(2-ethylhexyl)carbamimidoyl]carbamimidoyl]amino]hexyl]carbamimidoyl]guanidine		1 %	581.72

Compounds ID, Trivial name, IUPAC name, Structure, cytotoxicity and Molecular weight were provided by the MMV. Cytotoxicity values represent the viable percentage of the mammalian cells at 100 μM drug concentration. *MW- molecular weight.

### 3.6 Fungicidal concentration

Most compounds did not inhibit the growth of the *C. auris* in subsequent plating on SDA up to 10 μM concentration. These compounds were eberconazole, alexidine, AN-2718, carbendazim, amorolfine, MMV1782212, TSC-INH, MMV1782221,MMV1782218 and olorofim. However, few compounds inhibited *C. auris* on subsequent plating such as isavuconazonium (≥4 μM), MMV1634386 (≥2 μM), terbinafine (≥5 μM), MMV1782110 (≥2 μM) and abafungin (≥8 μM). Compounds like eberconazole, MMV1634386 and amorolfine produced no visible colonies of *C. albicans* in antibiotic free SDA plates from 4μMconcentration. Ketoconazole and terbinafine showed fungicidal effect against *C. albicans* at a concentration 5 μM.Luliconazole exhibited highest MFC of 8 μM. No fungicidal activity was observed in case of *A. niger,* up to 10 μM concentration (Table 3).

### 3.7 Mode of antifungal action

Fungicidal and fungistatic modes of action of the drugs were determined by the MFC:MIC ratio. MMV1782110 showed fungicidal effect on *C. auris,* and few drugs exhibited fungistatic action towards *C. auris* such as isavuconazonium, MMV1634386, terbinafine and abafungin. Other compounds that inhibited *C. auris* exhibited more than, or equal to, 1 MFC:MIC ratio, despite the MIC of the compounds having been >10 μM and >10 μM of MFC, respectively. Eberconazole, amorolfine and luliconazole were potential fungicidal compounds against *C. albicans.* MMV1634386 and ketoconazole were found to be fungistatic on *C. albicans*. Alexidine, the only compound that inhibited *A. niger*, also turned out to be fungistatic (Table 3.). Thus, these compounds were considered as potential compounds in our study.

## 4 Discussion

MDR *Pseudomonas* spp. (20.5 %) [23]and *Acinetobacter* spp. (58.51 %) [24]infections are often associated with high mortality and morbidity in Asia. In India, a retrospective study conducted on bloodstream infections showed that 29.9 % (32/107) of the *P. aeruginosa* population was MDR, with 68.8 % (22/32) of mortality, whereas MDR *A. baumannii* resistant to carbapenem was 80.4 % (37/46), with a mortality rate of 67.6 % (25/37) [25]. *Candida* spp. is the primary causative agent of fungal diseases, while *Aspergillus* spp. acting as the second most-common cause of hospital acquired fungal infections [26]. However, emergence of resistance to one or more groups of antifungals aggravated the treatment for patients with primary or secondary fungal infections [27]. Factors that incite the discovery of new drugs are: (1) the health sector being in need of new drugs to treat invasive bacterial or fungal pathogens; and (2) the rapid development and spread of resistance among these pathogens. Several research laboratories explored the application of MMV compounds on pathogens causing tuberculosis, schistosomiasis and other parasite diseases [28]. In this study, we screened the 201 antibacterial and 46 antifungal compounds from the MMV Pandemic Response Box to identify novel activity effective against MDR *A. baumannii* and *P. aeruginosa* bacterial clinical isolates as well as MDR *C. auris, C. albicans* and *A. niger* fungal pathogen isolates from the patients. The drug candidates were designated as potential inhibitory compounds if they showed high percentage of inhibition, low MIC values, ability to reduce persister viability in the assays followed by comparison with cytotoxicity values (provided by MMV). Cytotoxicity values were not considered into account while selecting the compounds for the assays.

In our study, 29 compounds at 10 μM inhibited *A. baumannii*, with nine of those compounds such as determined to be effective. Samoan et al. screened Pandemic Response Box compounds, too, and identified antibacterial activity against *A. baumannii,* ATCC strain (19606 and BC-5) at 50 μM [29] and *P. aeruginosa*PAO1 at 20 μM, respectively [30]. Thirty pandemic response box compounds inhibited ATCC *A. baumannii*, and eleven compounds inhibited *P. aeruginosa* PAO1, respectively [29,30]. It should be noted that the compounds, which inhibited the ATCC strain of *A.* baumannii in a previous study, were not inhibiting the clinical field isolate utilized in the current study [29]. Despite showing robust activity in preliminary testing against both clinical pathogens, twenty compounds failed to reduce the bacterial survival on subsequent plating onto LB agar in the persister assay. The variability of the results observed amongst drug resistant strains and sensitive strains, spotlights the limiting factor on the compound efficacy towards different strains of same bacterial species that could poses pandemic potential.

MMV1634390 (methyl N-[[3-(ethylcarbamoylamino)-5-(2-methylpyridin-4-yl)isoquinolin-8yl[methyl]carbamate) exhibited a complete bactericidal effect against *A. baumannii,* as evidenced by absence of bacterial colonies in the persister assay. Three compounds, MMV1578568(gepotidacin), MMV1578574 (eravacycline) and MMV1578566 (epetraborole) were found to be effective against *P. aeruginosa,* with more than 90 % of inhibition producing few persister colonies. Hit compounds in our study such as MMV1578568 (gepotidacin), MMV1578574 (eravacycline) and MMV1578566 (epetraborole) on *P. aeruginosa* produced a persister frequency of 1–6.3 × 10^1^ ± 10^1^ CFU/ml after compound treatment (Table 1). It is known that *A. baumannii* and *P. aeruginosa* pathogens have evolved to persist and develop resistance mechanisms like target modification, increased activity of efflux pumps (RND-type), and porin alterations (Opr) under drug pressure [31]. Gepotidacin (MMV1578568), a novel triazaacenaphthylene class antibiotic that functions as a type IIA topoisomerase inhibitor [32]. Phase II (NCT02045797)clinical trial to assess safety, tolerability and efficacy of gepotidacin in study participants with gram-positive bacteria skin infections has been completed [33].

MMV1580854, MMV1579788, MMV1578574 (eravacycline) and MMV1578566 (epetraborole) (Table 1) exhibited inhibition against both *A. baumannii* (≥80 % of growth inhibition) and *P. aeruginosa* (>50 % of growth inhibition). Eravacycline is a broad-spectrum antibiotic that is effective against both aerobic and anaerobic Gram-positive and Gram-negative bacteria. The basic mechanism of action of eravacycline is the blockage of the incorporation of amino acids into the elongating peptide chain by binding with 30S ribosomal subunits, which results in impaired protein synthesis. Similarly to tigecycline, eravacycline is unaffected by the resistance mechanism of bacteria such as acquired efflux systems and ribosomal protection proteins [34]. In our investigation, eravacycline was found to reduce the growth of *A. baumannii* and *P. aeruginosa* by 92 % and 98 %, respectively. Even though, eravacycline being immune to the development of resistance among bacterial pathogens, improper usage of these novel drug candidate during field use in real world settings may act as resistance promoting factor and studies necessitating further research. Epetraborole is a novel boron-containing antibiotic that targets leucyl-tRNA synthetase; and is effective against Gram-negative bacteria by evading efflux mechanisms. Epetraborole is found to be active against Gram-negative bacteria including *NDM-1, TEM-, OXA-, CTX-,* and *KPC-*carbapenemase-producing *Enterobacteriacae* (*K. pneumoniae, E. coli, P. aeruginosa, A. baumannii* and *P. mirabilis*) [35]. In our study, epetraborole inhibited 92 % and 97 % of *A. baumannii* and *P. aeruginosa*, respectively. Epetraborole was also found to be a potent inhibitor of *Mycobacterium abscessus* [36]. Although the mechanism of inhibition of both MMV1580854 and MMV1579788 is unclear, and information regarding the functional properties of the drugs is not readily available, these compounds were active against both clinical pathogens in our study [37,38]. MMV1580854 and MMV1579788 inhibited *A. baumannii* (96 % and 84 % of inhibition) and *P. aeruginosa* (53 % and 93 % of inhibition). The cytotoxicity of these compounds was estimated to be reasonable, with a cell survival rate above 70 % for the compounds at 100 μM concentration [18]. The synergistic effects of eravacycline with other drugs like ceftazidime, imepenem and colistin are studied, among which the combination with ceftazidime proved successful against carbapenem resistant *A. baumannii* isolates [39]. Epetraborole in combination with norvaline improved the treatment efficacy against *Mycobacterium* spp. [40]. The respective modes of action of these drugs as mentioned in this context were crucial for evaluating the efficacy. While some of the compounds such as epetraborole and eravacycline have broad spectrum activity against the majority of gram-negative bacteria, further research is needed to determine the spectrum range of other MMV pandemic response box compounds.

During initial screening for fungal pathogens, 15 compounds inhibited *C. auris*, six compounds inhibited *C. albicans*, and one inhibited *A. niger.* All the inhibitory compounds were able to produce more than 90 % of growth inhibition of all three fungal strains. Few noteworthy compounds such as isavuconazonium (0.5 μM), MMV1634386 (0.5 μM), terbinafine (2 μM), MMV1782110 (1 μM) and abafungin (3 μM) were found to possess antifungal activity on *C. auris* at lower MIC. Other compounds that exhibited inhibition of *C. auris* at minimum inhibitory concentration more or equal to 10 μM were not considered effective antifungal compounds. MFC of these compounds were also more than MIC concentration suggesting higher dosage required. Abovementioned compounds were fungistatic towards *C. auris*, excluding MMV1782110; with a MFC: MIC ratio of 2, demonstrating fungicidal mode of action. *C. albicans* responded to the hit compounds at lower MIC. Fungistatic action towards *C. albicans* was displayed by MMV1634386, ketoconazole and terbinafine, whereas eberconazole, amorolfine and luliconazole exerted fungicidal effects. Alexidine was the only hit compound that demonstrated lower MIC (3 μM) but higher MFC (>10 μM) against *A. niger*. Oteseconazole and terbinafine were identified to be potential antifungal agents against both *Candida* species; however, compound MMV1782110 showed better fungicidal efficacy against *C. auris.* Cytotoxicity of the compounds were already provided by MMV [18].

MMV1634386 or oteseconazole ((2R)-2-(2,4-difluorophenyl)-1,1-difluoro-3-(tetrazol-1-yl)-1-[5-[4-(2,2,2-trifluoroethoxy)phenyl]pyridin-2-yl]propan-2-ol) is an azole that targets 14α demethylase or CYP51. The demethylase enzyme plays an important role in the formation of ergosterol responsible for cell membrane integrity [41]. Oteseconazole demonstrated potent *invitro* activity against azole resistant *Candida* species [42]. In a recent phase 3 randomized, double-blind clinical trial, participants having acute vulvovaginal candidiasis (VVC) (n = 219)with recurrence were treated with oteseconazole and compared with fluconazole treated control group. Rate VVC resolution was similar between oteseconazole and fluconazole (93.5 % and 95.8 %). Oteseconazole showed a better result in controlling recurrence by week 50 when compared to fluconazole (5.1 % vs. 42.2 %) [43]. In contrast to previous generations of azoles, oteseconazole has excellent selectivity towards CYP51 and reduced interaction with human cytochrome P450s because of the tetrazole moiety [41], whereas the earlier-generation drugs like ketoconazole and fluconazole have imidazole and triazole moieties that tend to have much higher human cytochrome interaction, resulting in drug-drug interaction [41]. Disadvantage of the drug is the embryo-fetal toxicity affecting the female reproductive potential. The drug was approved in April 2022 by the US Food and Drug Administration (FDA) [41]. In our study, oteseconazole produced inhibition at lower MIC towards test *Candida* strains.

Terbinafine is an allylamine derivative that targets squalene epoxidase (squalene monooxygenase),involved in the formation of ergosterol (squalene 2,3-epoxidase > lanosterol > ergosterol) in cell wall synthesis of fungi, with short duration of therapy as an advantage [44]. Due to the lipophilic nature of terbinafine, it tends to accumulate in fatty sites like adipose tissue, and nails [45]. Terbinafine in our study manifested fungistatic action on both *Candida* species although the activity was more towards fungicidal mode based on MFC:MIC ratio.

MMV1782110 or 3-(1,3-benzodioxol-5-yl)-N-[[4-(2-fluorophenyl) phenyl]methyl] propanamide is a novel compound with scanty information available [46]. The compound was subjected to antibacterial activity against *P. aeruginosa* and *E. coli, against which* it exhibited a MIC of 20 μM [46]. Alexidine or (1-(2-ethylhexyl)-3-[N-[6-[[N-[N-(2-ethylhexyl)carbamimidoyl] carbamimidoyl]amino] hexyl] carbamimidoyl]guanidine) is an amphipathic bisbiguanide that contains two (2-ethylhexyl)guanide units linked by hexamethylene [47]. Alexidine is an anticancer drug that inhibits mitochondrial phosphatase PTPMT1 in mammalian cells causing mitochondrial apoptosis [48]. It was also reported that alexidine displayed potential activity against clinically significant fluconazole resistant *C. albicans, C. glabrata, C. parapsilosis and C. auris.* Furthermore, this compound completely inhibited planktonic growth and biofilm formation of the filamentous fungi like *A. fumigatus* [49]. The compound completely eradicated filament formation of *A. niger* up from a concentration of 3 μM in our assay.

In this study, some of the antibacterial and antifungal compounds in the study demonstrated potential inhibitory activity against the clinical isolates. However, the efficacy of these compounds against the target pathogens in pre-clinical and clinical models has to be investigated to progress further. Furthermore, it should be taken into consideration that these compounds were not giving the same inhibitory activity against the entire spectrum of strains belonging to the same species. Analyzing each of these substances separately on a wide range of strains from various origins and representing different geographical regions would yield valuable information. Different phases of clinical trials of some compounds were underway to assess the safety, side effects and efficacy in various infections models. With limited data of pharmacokinetics and pharmacodynamics of the potent compounds, more investigation and clinical trials are in demand. Discovery of new drugs with proper stewardship and surveillance on the usage of these drugs, preventing overexploitation, is inevitable to mitigate the drug resistance. Moreover, these pathogens are silent-pandemic pathogens and drug resistance in these pathogens adds more complexity in eliminating these pathogens using individual drug than combination therapies. Screening of MMV novel drugs with existing high end drug molecules could yield valuable information. Drug resistance in pathogens were discovered shortly after the usage first antibiotic and been an unsettling threat ever since, long term sustainability of the new drugs for treatment of these drug resistant pathogens is questionable. This also emphasizes the need for the alternate therapeutic methods, including vaccines, early diagnostics, immune boosters and most importantly non-pharmaceutical interventions, that ensure safe and effective combat strategies against drug resistance.

In conclusion, eravacycline and epetraborole were the two most active broad-spectrum compounds against clinical field isolates of *A. baumannii* and *P. aeruginosa.* Phase III (NCT01844856) and phase II (NCT01381549) clinical trials for eravacycline and epetraborole were conducted, respectively [39,40]. Scanty information on two other compounds (MMV1580854 and MMV1579788) might be a limiting factor for understanding the mode of action. Furthermore, enzyme kinetics, pharmacokinetics/pharmacodynamics studies of these two drugs might reveal better insights of the compounds. Similarly, we identified few potent antifungal compounds inhibiting the invasive nosocomial fungal pathogens like *Candida* spp. and *Aspergillus* spp. Oteseconazole and terbinafine were effective antifungal candidates for *C. albicans* and *C. auris*. Alexidine was able to inhibit *A. niger.* MMV1782110 showed excellent inhibition of *C. auris*. The current study emphasizes the importance of drug discovery to mitigate drug resistance and fosters future investigations for alternate approaches.

## Funding statement

The study was supported by the Indian Council of Medical Research (ICMR), New Delhi, India, extramural core research grant ID- AMR/ADHOC/242/2020-ECD-II.

## CRediT authorship contribution statement

**Seshan Sivasankar:** Writing – review & editing, Writing – original draft, Methodology. **Appalaraju Boppe:** Writing – review & editing, Methodology. **Martin Peter Grobusch:** Writing – review & editing. **Sankarganesh Jeyaraj:** Writing – review & editing, Methodology, Investigation, Conceptualization.

## Declaration of competing interest

The authors declare that they have no known competing financial interests or personal relationships that could have appeared to influence the work reported in this paper.

## References

[1] Murray C.J.L., Ikuta K.S., Sharara F., Swetschinski L., Aguilar G.R., Gray A. (2022). Global burden of bacterial antimicrobial resistance in 2019: a systematic analysis. Lancet.

[2] Fisher M.C., Alastruey-Izquierdo A., Berman J., Bicanic T., Bignell E.M., Bowyer P. (2022). Tackling the emerging threat of antifungal resistance to human health. Nat Rev Microbiol.

[3] Pang Z., Raudonis R., Glick B.R., Lin T.-J., Cheng Z. (2019). Antibiotic resistance in Pseudomonas aeruginosa: mechanisms and alternative therapeutic strategies. Biotechnol Adv.

[4] Kyriakidis I., Vasileiou E., Pana Z.D., Tragiannidis A. (2021). Acinetobacter baumannii antibiotic resistance mechanisms. Pathogens.

[5] Carvalheira A., Silva J., Teixeira P. (2021). Acinetobacter spp. in food and drinking water - a review. Food Microbiol.

[6] Martis N., Leroy S., Blanc V. (2014). Colistin in multi-drug resistant Pseudomonas aeruginosa blood-stream infections: a narrative review for the clinician. J Infect.

[7] Lim L.M., Ly N., Anderson D., Yang J.C., Macander L., Jarkowski A. (2010). Resurgence of colistin: a review of resistance, toxicity, pharmacodynamics, and dosing. Pharmacotherapy.

[8] Azimi L., Lari A.R. (2019). Colistin-resistant Pseudomonas aeruginosa clinical strains with defective biofilm formation. GMS Hyg Infect Control.

[9] WHO publishes list of bacteria for which new antibiotics are urgently needed n.d. https://www.who.int/news/item/27-02-2017-who-publishes-list-of-bacteria-for-which-new-antibiotics-are-urgently-needed (accessed June 16, 2023).

[10] Gudlaugsson O., Gillespie S., Lee K., Vande Berg J., Hu J., Messer S. (2003). Attributable mortality of nosocomial candidemia, revisited. Clin Infect Dis.

[11] Tong X., Liu T., Jiang K., Wang D., Liu S., Wang Y. (2021). Clinical characteristics and prognostic risk factors of patients with proven invasive pulmonary aspergillosis: a single-institution retrospective study. Front Med.

[12] Bassetti M., Vena A., Bouza E., Peghin M., Muñoz P., Righi E. (2020). Antifungal susceptibility testing in Candida, Aspergillus and Cryptococcus infections: are the MICs useful for clinicians?. Clin Microbiol Infection.

[13] Friedman D.Z.P., Schwartz I.S. (2019). Emerging fungal infections: new patients, new patterns, and new pathogens. J Fungi (Basel).

[14] Wall G., Herrera N., Lopez-Ribot J.L. (2019). Repositionable compounds with antifungal activity against Multidrug resistant Candida auris identified in the Medicines for Malaria venture's pathogen box. J Fungi (Basel).

[15] (2022). The biggest antibiotic-resistant threats in the U.S.

[16] Person A.K., Chudgar S.M., Norton B.L., Tong B.C., Stout J.E. (2010). Aspergillus Niger: an unusual cause of invasive pulmonary aspergillosis. J Med Microbiol.

[17] Jacobs S.E., Jacobs J.L., Dennis E.K., Taimur S., Rana M., Patel D. (2022). Candida auris pan-drug-resistant to four classes of antifungal agents. Antimicrob Agents Chemother.

[18] About the Pandemic Response Box. Medicines for Malaria Venture n.d. https://www.mmv.org/mmv-open/pandemic-response-box/about-pandemic-response-box (accessed March 13, 2023).

[19] M07: Dilution AST for Aerobically Grown Bacteria - CLSI. Clinical & Laboratory Standards Institute n.d. https://clsi.org/standards/products/microbiology/documents/m07/(accessed March 13, 2023).

[20] Simner P.J., Bergman Y., Trejo M., Roberts A.A., Marayan R., Tekle T. (2019). Two-site evaluation of the colistin broth Disk elution test to determine colistin in vitro activity against gram-negative bacilli. J Clin Microbiol.

[21] Bardet L., Rolain J.-M. (2018). Development of new tools to detect colistin-resistance among enterobacteriaceae strains. Can J Infect Dis Med Microbiol.

[22] Hafidh R.R., Abdulamir A.S., Vern L.S., Abu Bakar F., Abas F., Jahanshiri F. (2011). Inhibition of growth of highly resistant bacterial and fungal pathogens by a natural product. Open Microbiol J.

[23] Lee Y.-L., Ko W.-C., Hsueh P.-R. (2022). Geographic patterns of carbapenem-resistant Pseudomonas aeruginosa in the asia-pacific region: results from the antimicrobial testing leadership and surveillance (ATLAS) program, 2015-2019. Antimicrob Agents Chemother.

[24] Teerawattanapong N., Panich P., Kulpokin D., Ranong S.N., Kongpakwattana K., Saksinanon A. (2018). A systematic review of the burden of multidrug-resistant healthcare-associated infections among intensive care unit patients in southeast Asia: the rise of multidrug-resistant acinetobacter baumannii. Infect Control Hosp Epidemiol.

[25] Balkhair A., Saadi K.A., Adawi B.A. (2023). Epidemiology and mortality outcome of carbapenem- and colistin-resistant Klebsiella pneumoniae, Escherichia coli, Acinetobacter baumannii, and Pseudomonas aeruginosa bloodstream infections. IJID Regions.

[26] Perlroth J., Choi B., Spellberg B. (2007). Nosocomial fungal infections: epidemiology, diagnosis, and treatment. Med Mycol.

[27] Arendrup M.C. (2014). Update on antifungal resistance in Aspergillus and Candida. Clin Microbiol Infection.

[28] Duffy S., Sykes M.L., Jones A.J., Shelper T.B., Simpson M., Lang R. (2017). Screening the Medicines for Malaria venture pathogen box across multiple pathogens reclassifies starting points for open-source drug discovery. Antimicrob Agents Chemother.

[29] Upmanyu K., Haq Q.M.R., Singh R. (2023). Novel antibacterial and antibiofilm compounds identified from open source library screening against priority pathogen acinetobacter baumannii. Int J Infect Dis.

[30] Macho M., Saha S., Konert G., Banerjee A., Ewe D., Hrouzek P. (2022). Screening of the Medicines for Malaria venture pandemic response box for discovery of antivirulent drug against Pseudomonas aeruginosa. Microbiol Spectr.

[31] Rice L.B. (2006). Challenges in identifying new antimicrobial agents effective for treating infections with Acinetobacter baumannii and Pseudomonas aeruginosa. Clin Infect Dis.

[32] O'Riordan W., Tiffany C., Scangarella-Oman N., Perry C., Hossain M., Ashton T. (2017). Efficacy, safety, and tolerability of gepotidacin (GSK2140944) in the treatment of patients with suspected or confirmed gram-positive acute bacterial skin and skin structure infections. Antimicrob Agents Chemother.

[33] GlaxoSmithKline. A Phase II (2017).

[34] Scott L.J. (2019). Eravacycline: a review in complicated intra-abdominal infections. Drugs.

[35] Hernandez V., Crépin T., Palencia A., Cusack S., Akama T., Baker S.J. (2013). Discovery of a novel class of boron-based antibacterials with activity against gram-negative bacteria. Antimicrob Agents Chemother.

[36] Kim T., Hanh B.-T.-B., Heo B., Quang N., Park Y., Shin J. (2021). A screening of the MMV pandemic response box reveals epetraborole as A new potent inhibitor against Mycobacterium abscessus. Int J Mol Sci.

[37] PubChem. 2-(2-Aminopyridin-3yloxy)-5-ethyl-4-fluorophenol n.d. https://pubchem.ncbi.nlm.nih.gov/compound/59191218 (accessed March 13, 2023).

[38] PubChem. 5-bromo-3,3-dimethyl-1H-pyrrolo[2,3-b]pyridin-2-one;deuterium monohydride;(E)-3-(3,3-dimethyl-2-oxo-1H-pyrrolo[2,3-b]pyridin-5-yl)-N-methyl-N-[(3-methyl-1-benzofuran-2-yl)methyl]prop-2-enamide;N-methyl-N-[(3-methyl-1-benzofuran-2-yl)methyl]prop-2-enamide;hydroiodide n.d. https://pubchem.ncbi.nlm.nih.gov/compound/162168788 (accessed March 13, 2023).

[39] Li Y., Cui L., Xue F., Wang Q., Zheng B. (2022). Synergism of eravacycline combined with other antimicrobial agents against carbapenem-resistant Enterobacteriaceae and Acinetobacter baumannii. Journal of Global Antimicrobial Resistance.

[40] Sullivan J.R., Lupien A., Kalthoff E., Hamela C., Taylor L., Munro K.A. (2021). Efficacy of epetraborole against Mycobacterium abscessus is increased with norvaline. PLoS Pathog.

[41] PubChem. Oteseconazole n.d. https://pubchem.ncbi.nlm.nih.gov/compound/77050711.

[42] Logan A., Wolfe A., Williamson J.C. (2022). Antifungal resistance and the role of new therapeutic agents. Curr Infect Dis Rep.

[43] Martens M.G., Maximos B., Degenhardt T., Person K., Curelop S., Ghannoum M. (2022). Phase 3 study evaluating the safety and efficacy of oteseconazole in the treatment of recurrent vulvovaginal candidiasis and acute vulvovaginal candidiasis infections. Am J Obstet Gynecol.

[44] Gokhale V.M., Kulkarni V.M. (2000). Understanding the antifungal activity of terbinafine analogues using quantitative structure–activity relationship (qsar) models. Bioorg Med Chem.

[45] Kanakapura B., Penmatsa V.K. (2016). Analytical methods for determination of terbinafine hydrochloride in pharmaceuticals and biological materials. Journal of Pharmaceutical Analysis.

[46] PubChem. 3-(1,3-benzodioxol-5-yl)-N-[[4-(2-fluorophenyl)phenyl]methyl]propanamide n.d. https://pubchem.ncbi.nlm.nih.gov/compound/137648729.

[47] PubChem. Alexidine n.d. https://pubchem.ncbi.nlm.nih.gov/compound/2090.

[48] Doughty-Shenton D., Joseph J.D., Zhang J., Pagliarini D.J., Kim Y., Lu D. (2010). Pharmacological targeting of the mitochondrial phosphatase PTPMT1. J Pharmacol Exp Ther.

[49] Mamouei Z., Alqarihi A., Singh S., Xu S., Mansour M.K., Ibrahim A.S. (2018). Alexidine dihydrochloride has broad-spectrum activities against diverse fungal pathogens. mSphere.

