# Supplementary material for: Evaluation of MMV Pandemic Response Box compounds to identify potent compounds against clinically relevant bacterial and fungal clinical isolates in vitro
Source: New Microbes New Infect. 2024 Jun 20;60-61:101444. doi: 10.1016/j.nmni.2024.101444 (PMC11261442; doi:10.1016/j.nmni.2024.101444)
Supplement: Multimedia component 1 [file mmc1.docx]

**Supplementary Table 1.** Antibiogram analysis of *A. baumannii* and *P. aeruginosa* test isolates using Vitek2®*.**S- sensitive, R- resistant, NA- not applicable.

| Antibiotics | *Acinetobacter baumannii* | *Pseudomonas aeruginosa* |
| --- | --- | --- |
| Aztreonam | NA | S |
| Netilmicin | S | R |
| Ceftazidime | R | R |
| Ceftazidime-Avibactam | S | R |
| Cefoperazone+Sulbactam | S | R |
| Meropenem | S | R |
| Piperacillin+Tazobactam | S | R |
| Amikacin | S | R |
| Colistin | R-8 µg/ml | R-8µg/ml |
| Tobramycin | NA | R |
| Cefepime | S | R |
| Imipenem | S | R |
| Levofloxacin | S | R |
| Piperacillin | S | R |
| Ciprofloxacin | S | R |
| Gentamicin | S | R |
| Ampicillin+Sulbactam | S | NA |
| Co - Trimoxazole | S | NA |
| Minocycline | S | NA |
| Tetracycline | S | NA |
| Tigecycline | S | NA |
| Ofloxacin | S | NA |
| Doxycycline | S | NA |

**Supplementary Table2.** Antibiogram analysis of fungal isolates usingVitek2®*.**S- sensitive, R- resistant, NA- not applicable.

| Antibiotics | *C. albicans* | *C. auris* | *A.niger* |
| --- | --- | --- | --- |
| Amphotericin B | S | R | NA |
| Itraconazole | S | S | NA |
| Ketoconazole | R | S | NA |
| Fluconaozole | R | R | NA |
| Voriconazole | S | S | NA |
| Clotrimazole | R | S | NA |
